# Microvascular dysfunction in ankylosing spondylitis is associated with disease activity and is improved by anti-TNF treatment

**DOI:** 10.1038/s41598-018-31550-y

**Published:** 2018-09-04

**Authors:** Bogdan Batko, Pawel Maga, Karol Urbanski, Natalia Ryszawa-Mrozek, Agata Schramm-Luc, Mateusz Koziej, Tomasz Mikolajczyk, Eilidh McGinnigle, Marta Czesnikiewicz-Guzik, Piotr Ceranowicz, Tomasz J. Guzik

**Affiliations:** 1 Department of Rheumatology, J Dietl Hospital, Krakow, Poland; 2 Department of Angiology, II Chair of Internal Medicine, Jagiellonian University School of Medicine, Krakow, Poland; 3 Department of Internal and Agricultural Medicine, Jagiellonian University School of Medicine, Krakow, Poland; 4 Department of Anatomy, Jagiellonian University School of Medicine, Krakow, Poland; 5 BHF Centre of Research Excellence, Institute of Cardiovascular and Medical Sciences, University of Glasgow, Glasgow, United Kingdom; 6 Institute of Infection Immunity and Inflammation, University of Glasgow, Glasgow, United Kingdom; 7 Department of Physiology, Jagiellonian University School of Medicine, Krakow, Poland

## Abstract

Ankylosing spondylitis (AS) is associated with high cardiovascular morbidity and mortality. Recent studies indicate that microvascular dysfunction may underlie cardiovascular risk in AS. We hypothesized, that microvascular morphology and dysfunction is linked to AS activity and is modifiable by TNF-α inhibitor (TNFi) treatment. Functional Laser Doppler Flowmetry with post-occlusive reactive hyperemia, and structural nailfold capillaroscopy were performed in 54 patients with AS and 28 matched controls. Active AS was diagnosed based on BASDAI ≥ 4 (n = 37). Effects of 3-month TNFi on microcirculation in active AS were studied. AS was associated with prolonged time to peak hyperemia compared to healthy controls. High disease activity was associated with increased time to peak hyperemia and decreased peak hyperemia when compared to patients with inactive AS. In capillaroscopy, AS was associated with morphological abnormalities indicating increased neoangiogenesis and pericapillary edema compared to controls. Microvascular function improved following 3 months of TNFi in reference to basal flow as well as post-occlusive parameters. TNFi reduced pericapillary edema, while other parameters of capillary morphology remained unchanged. Microvascular dysfunction and capillary neovascular formation are associated with disease activity of AS. Anti-TNF-α treatment may restore microcirculation function and capillary edema but does not modify microvascular structural parameters.

## Introduction

Ankylosing spondylitis (AS) is associated with elevated cardiovascular (CV) risk and increased mortality^1–4^, as an independent CVD risk factor. Registry data suggest a 30–50% increased risk of CV events compared to general population^5^. The mechanisms of this link are not thoroughly investigated but are thought to be related to dysregulated CV risk factors profile as well as directly to inflammatory mechanisms of AS. Endothelial dysfunction is a key mechanism of increased CVD risk^6^ with conventional causes such as hypertension, diabetes mellitus (DM), smoking and hypercholesterolemia^7–9^, but can also be induced by inflammation^10^. While the majority of studies have focused on the dysfunction of large conduit vessels in AS^11,12^, less is known in relation to microvascular dysfunction in this disease. While vascular dysfunction in conduit vessels underlies atherosclerosis, microvascular dysfunction serves as a key mechanism for hypertension, cognitive dysfunction and heart failure via effects in *vasa vasorum* contributing to perivascular inflammation – an early feature of vascular disease^13–15^. Therefore, characterization of microcirculation in AS and its links to AS disease activity, is of primary importance. Skin microvasculature offers a perfect model to study arteriole function and capillary morphology^16^. So far only one, recent study has shown that NO-mediated, acetylcholine induced vasorelaxation in microvessells is impaired in AS and may improve with therapy^17^. We hypothesized that microvascular morphology and function in patients with AS is linked to disease activity and is modifiable by TNF-α inhibitor (TNFi) treatment, reducing disease activity. Presence of microvascular dysfunction has been recently identified in a smaller and mixed populations of patients with inflammatory joint diseases^17,18^. It, however, remains unclear whether this relationship is caused by prevalence of classical CVD risk factors or is linked to AS specific inflammatory factors. It is unclear how such dysfunction is linked to AS disease activity. Finally, microvascular morphological changes have not been assessed so far in this context. Therefore, using physiological stimulus – hyperemia and Laser Doppler Flowmetry (LDF), in a well powered analysis, we have confirmed presence of microvascular dysfunction in a low classical CVD risk population, and observed a clear relationship between functional microvascular impairment, morphological changes and AS activity. Moreover, we demonstrated that functional impairment and capillary edema can be reversed by anti-TNF treatment, suggesting a key role of this drug therapy in microvascular dysfunction in patients with highly active AS. Thus, simple tools for the measurement of microvascular function in the skin, available in typical outpatient setting, may give a valuable insight into microvascular function and structure and can be monitored in relation to its effects on cardiovascular risk.

## Results

### Microvascular function and morphology in Ankylosing Spondylitis

Microcirculation parameters studied using Laser Doppler Flowmetry (LDF) with Post-Occlusive Reactive Hyperemia (PORH) were impaired in AS patients compared to healthy controls. The groups were relatively young [40.0 (34–48) and 43.5 (39–48) years old respectively], matched for major clinical factors affecting microvascular function and their occurrence was low when compared to general population (hypertension <15%) or absent (diabetes mellitus, thyroid dysfunction, Raynaud’s phenomenon, and previous cardiovascular events) (Table 1). Time to peak hyperemia was almost two-fold longer in comparison to healthy controls [9.4 s (6.8–12.5) vs 5.2 s (3.8–6.1); p < 0,001] (Table 2). In capillaroscopy, AS was associated with an abnormal microvascular morphology, with increased occurrence of loops enlargements, bushy and coiled, and branched capillaries as well as pericapillary edema compared to healthy controls (20.4% vs 3.6%, p = 0.04; 75.9% vs. 21.4%, p < 0.001; 37% vs. 14.3%, p = 0.03; 46.3% vs. 3.6%, p < 0.001 respectively) (Table 3).

**Table 1:** Baseline characteristics of Ankylosing Spondylitis patients with active and inactive disease and healthy controls.

	All AS (n = 54)	Inactive (n = 17)	Active (n = 37)	P value Active vs Inactive	Healthy controls (n = 28)	P value AS vs Healthy controls
Age, years	40.0 (34–48)	41.0 (35–47)	40.0 (31–48)	0.79	43.5 (39–48)	0.24
Male, %	66.7	76.5	62.2	0.30	46.4	0.08
Body Mass Index	26.7 (22.3–29.3)	27.1 (22.8–30.0)	26.4 (22.0–28.9)	0.51	26.4 (24.4–28.9)	0.62
Smoking, %	20.4	17.7	21.6	1	17.9	0.79
Hypercholesterolemia,%	68.5	82.4	62.1	0.21	85.7	0.09
Hypertension, %	13.0	11.8	13.5	1	0	0.09
Disease duration, years	6.5 (2.0–12.5)	7.0 (3.0–12.0)	6.0 (2.0–13.0)	0.54	—	—
BASDAI	6.2 (3.8–7.2)	2.0 (1.6–3.2)	6.7 (6.0–7.7)	**<0**.**001**	—	—
VAS pain	64.0 (35.0–74.0)	23.0 (8.0–35.0)	70.0 (62.0–78.0)	**<0**.**001**	—	—
ASDAS – CRP	3.5 (2.0–4.2)	1.4 (1.0–1.9)	3.8 (3.4–4.3)	**<0**.**001**	—	—
ASDAS - ESR	3.3 (1.9–3.6)	1.4 (0.8–1.9)	3.4 (3.2–4.0)	**<0**.**001**	—	—
CRP, mg/dl	8.0 (3.2–14)	3.3 (1.8–4.3)	9.7 (6.1–20.6)	**<0**.**001**	1.4 (0.8–2.5)	**<0**.**001**
ESR, mm/h	12.1 (6.1–22.3)	6.1 (3.0–10.3)	17.4 (9.0–26.6)	**0**.**002**	—	—

Data are median (IQR). Mann-Whitney U test was used for comparing continuous variables. Chi-square test or Fisher exact test were used for dichotomous variables. AS – Ankylosing Spondylitis.

**Table 2:** Microvascular function parameters in patients with Ankylosing Spondylitis and healthy controls.

	Ankylosing Spondylitis (n = 54)	Healthy Controls (n = 28)	P value
Basal Flow [PU]	32.1 (18.9–63.1)	29.0 (24.6–35.5)	0.41
Peak hyperemia [PU]	98.2 (38.3–184.5)	96.7 (79.5–117.7)	0.75
Time to peak [s]	9.4 (6.8–12.5)	5.2 (3.8–6.1)	**<0**.**001**

Data are median (interquartile range). Mann-Whitney U test was used.

**Table 3:** Capillaroscopic parameters in patients with Ankylosing Spondylitis and healthy controls.

	Ankylosing Spondylitis (n = 54)	Healthy Controls (n = 28)	P value
Capillary disorganization	25.9%	21.4%	0.65
Loss of capillaries	22.2%	35.7%	0.19
Loop enlargements	20.4%	3.6%	**0**.**04**
Megacapillaries	0%	0%	—
Bushy and coiled capillaries	75.9%	21.4%	**<0**.**001**
Branched capillaries	37.0%	14.3%	**0**.**03**
Pericapillary edema	46.3%	3.6%	**<0**.**001**

The number and morphology were evaluated in Ankylosing Spondylitis patients and healthy controls. The percentages reflect the number of participants in which the mentioned structures were present. Chi-square test was used.

### Microvasculature, nailfold capillaroscopy and disease activity

**Figure 1:**
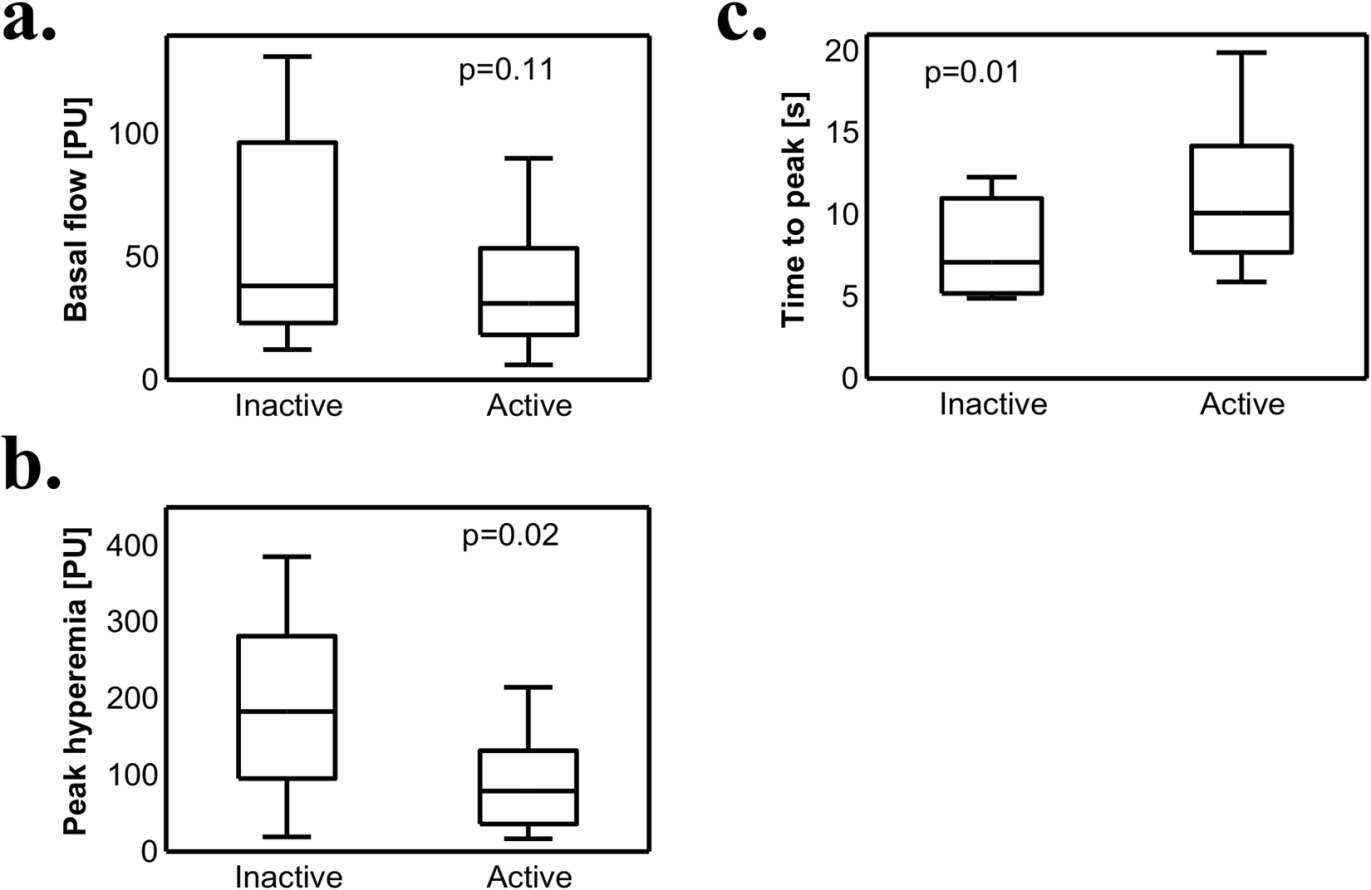
Disease activity and microvascular function. Basal flow (**a**), peak hyperemia (**b**) and time to peak hyperemia (**c**) were measured in patients with active (n = 37) and inactive (n = 17) disease based on BASDAI score. Mann-Whitney U test was used. Data are expressed as median, interquartile range (box), 10–90 percentile (whiskers).

To evaluate whether the observed differences are associated with disease activity we divided patients into 2 groups based on Bath Ankylosing Spondylitis Disease Activity Index (BASDAI). No differences in age, sex, body mass index (BMI), smoking status nor disease duration were observed between the groups (Table 1). Patients with active disease (BASDAI ≥ 4) had impaired microcirculation function in PORH compared to the inactive group (BASDAI < 4). They were characterized by decreased peak hyperemia [79.1PU (36.0–131.9) vs 183.1PU (95.5–281.7); p = 0.02] and increased time to peak [10.1 s (7.7–14.2) vs 7.1 s (5.2–11.0); p = 0.01). Basal flow was not influenced by disease activity [31.1PU (18.3–53.5) vs. 38.2PU (23.1–96.5); p = 0.11], (Fig. 1). No changes were observed in capillaroscopy between patients with active and inactive disease (p > 0.05) (see Supplementary Table S1).

### TNF-α inhibitor therapy and disease activity

TNF-α inhibitor treatment effectively reduced AS disease activity. The values of BASDAI, Visual Analog Scale (VAS) pain, Ankylosing Spondylitis Disease Activity score (ASDAS-CRP), and ASDAS-ESR were significantly lowered after the first 3 months of therapy [6.70 (6.0–8.0) to 3.20 (2.5–3.7), p < 0.001; 72.0 (65.0–78.0) to 23.0 (11.0–35.0), p < 0.001; 4.0 (3.5–4.3) to 1.8 (1.5–2.0), p < 0.001; 3.4 (3.3–3.9) to 1.4 (1.3–2.0), p < 0.001, respectively].

### TNF-α inhibitor therapy and microcirculation parameters

**Figure 2:**
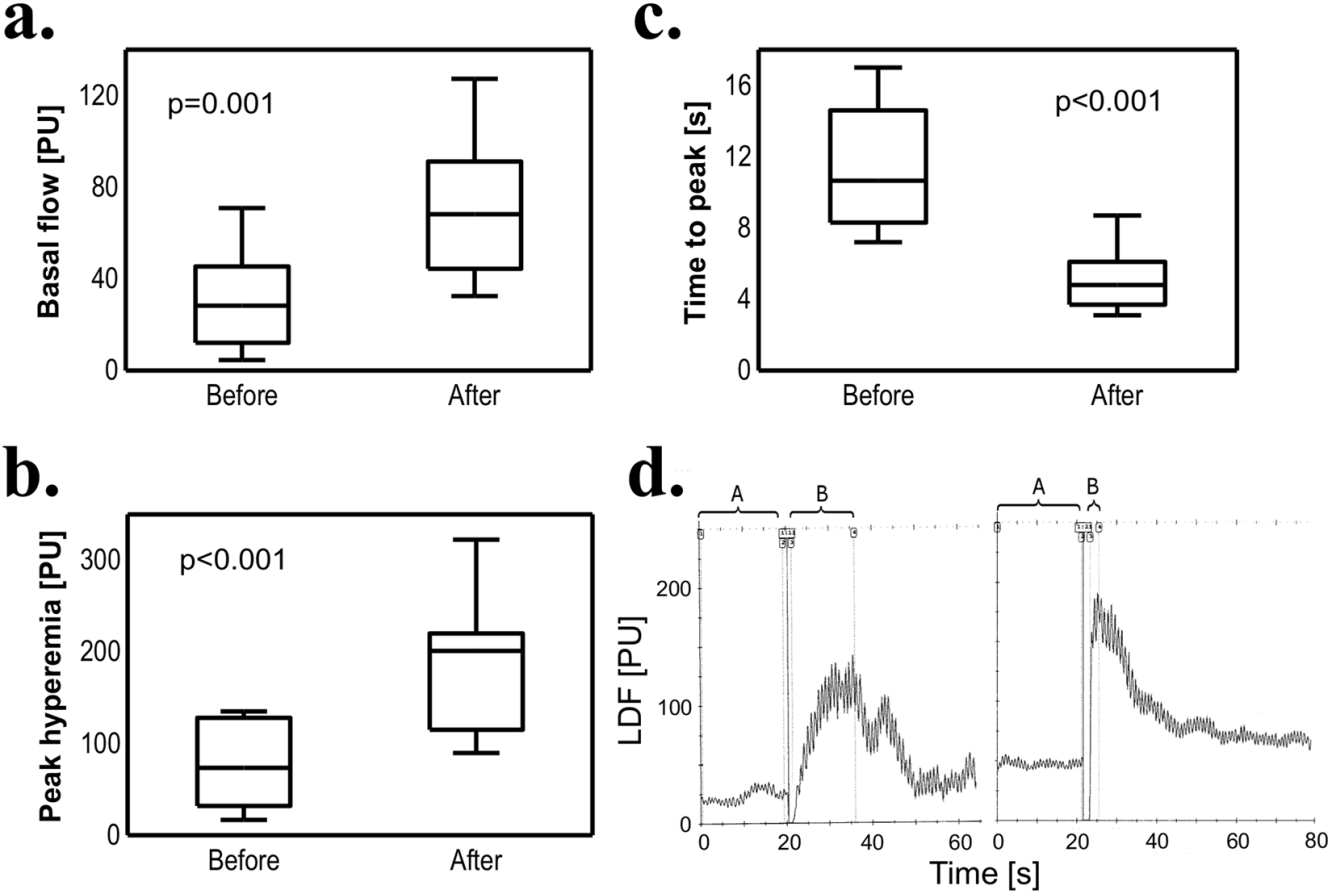
TNF-α inhibitor treatment and microvascular function. Basal flow (**a**), peak hyperemia (**b**) and time to peak hyperemia (**c**) were measured in patients before and 3 months after TNFi treatment (n = 22). (**d**) Sample test result of LDF and PORH: (left) patient before TNFi treatment, (right) after TNFi treatment: A - basal flow, B - PORH test – peak hyperemia after 5-minute occlusion. Wilcoxon signed-rank test was used. Data are expressed as median, interquartile range (box), 10–90 percentile (whiskers). TNFi – TNF-α inhibitor, LDF – Laser Doppler Flowmetry, PORH – Post-occlusive Reactive Hyperemia.

To evaluate the influence of treatment with TNF-α inhibitors on microcirculation function, we performed LDF before commencing and after 3 months of TNFi therapy. There was an improvement in all observed microcirculation parameters. Basal flow and peak hyperemia were significantly increased [28.3PU (12.1–45.4) vs. 68.2PU (44.4–91.2); p = 0.001 and 73.6PU (32.0–128.1) vs. 201.1PU (115.1–220.1); p < 0.001, respectively]. Meanwhile, time to peak was decreased in comparison to values obtained before biological treatment [10.7 s (8.3–14.6) vs. 4.8 s (3.7–6.1); p < 0.001]; (Fig. 2a–c). Example of the results of LDF with hyperemia test before and after biologic treatment is shown in Fig. 2d.

### TNF-α inhibitor therapy and nailfold capillaroscopy

TNF-α inhibitor treatment influenced only one capillaroscopy parameter. At 3 months of TNFi therapy, reduced pericapillary edema was noted (36.4% vs 4.5%; p = 0.02); (Table 4 and Supplementary Fig. S1). There was no change in other morphological parameters, such as the type of visible capillaries, capillary density and distribution.

**Table 4:** Capillaroscopic parameters before and after TNF-α inhibitor treatment.

	Before TNFi treatment	After TNFi treatment	P value
Capillary disorganization	27.3%	31.8%	1
Loss of capillaries	27.3%	13.6%	0.25
Loop enlargements	13.6%	18.2%	1
Megacapillaries	0%	0%	—
Bushy and coiled capillaries	86.4%	77.3%	0.62
Branched capillaries	31.8%	31.8%	1
Pericapillary edema	36.4%	4.5%	**0**.**02**

The number and morphology were evaluated before and 3 months after TNFi treatment (n = 22). The percents reflect the number of participants in which the mentioned structures were present. McNemar’s test was used. TNFi – TNF-α inhibitor.

## Discussion

We compared microvascular morphology and function in well matched cohorts of AS patients with differing levels of severity and healthy controls. Subjects matched for age, sex, BMI and major CVD risk factors (low traditional risk population). Our study demonstrates, that microvascular impairment in AS is associated with disease activity. Anti-TNF-α treatment restores microvascular function and diminishes peri-capillary edema assessed by capillaroscopy in patients with high disease activity. While impaired microvascular function has been shown recently by smaller studies using invasive methods^17^ or in mixed un-defined arthritis populations^18^, we have used appropriately powered population size of subjects with AS with low traditional CV risk. We also demonstrated that simple, non-invasive techniques, available in most rheumatology clinics may be useful in assessing microvascular status. Finally, we have shown that TNFi effects persist past the therapy initiation^17^, and are very clear at 3 month timepoint.

Several chronic inflammatory diseases are associated with increased cardiovascular risk^19^. Most previous studies have focused on rheumatoid arthritis (RA)^20^ and systemic lupus erythematosus (SLE)^21,22^. The data regarding spondyloarthropaties, including AS are limited, although CV mortality and morbidity increase is evident^23^. It is not clear however, if increased CV risk is caused by inflammatory disease activity or is secondary to traditional risk factors, such as dyslipidemia^24^ or hypertension^6^, that are often present in AS.

Numerous model and observational studies and more recently clinical randomized trials, such as CANTOS, indicate an important role of inflammation in mediating increased cardiovascular risk^25–27^. Inflammatory cells and mediators induce endothelial dysfunction^6^, subsequent plaque formation and plaque instability resulting in acute coronary syndromes. Thus, anti-inflammatory therapies may provide several protective mechanisms in vascular dysfunction in atherosclerosis^28^. A recent meta-analysis suggests that TNF-α inhibitors improve endothelial function in patients with Rheumatoid Arthritis^29^. Comparable effects in AS may be attributed to reducing systemic inflammation present in these two diseases. On the other hand, inflammation is less pronounced in spondyloarthropaties, so the effects may be weaker and more difficult to confirm. Studies of inflammatory mechanisms of cardiovascular risk focus on macrovascular dysfunction and disease. While this is important for acute MI development or ischemic strokes, numerous chronic conditions are associated with microvascular dysfunction, which may be a key mechanism for heart failure development, cognitive impairment and vascular dementia^30,31^. Moreover, it has been reported that microvascular dysfunction may lead to CV events even in the absence of obstructive epicardial coronary artery disease^32^. Therefore, identification of profoundly impaired microvascular flow mediated endothelial function and abnormalities within microvessel morphology may have numerous long term clinical consequences. Thanks to careful selection of study population without CAD or diabetes with low prevalence of other classical risk factors and matching for age, sex and BMI, we were able to assess effects particularly attributable to AS itself. This was further supported by links to disease activity and improvement with TNFi therapy. While the fact that we studied patients treated using different TNF-α inhibitors could be considered as limitation, it also suggests, that the observed effects are not limited to one biologic agent. The question remains whether the type of TNF-α inhibitor is of importance and affects the quantity and kind of changes in the microcirculation. This could be essential in the treatment choice for patients with high CVD risk and such large randomized studies will hopefully clarify this in the future.

We used much simpler methodology of assessment than used in a seminal study of van Eijk *et al*.^17^ Approach presented in this study is non-invasive, reproducible and poses little discomfort to the patient^33^. Thus, it is potentially valuable for vascular risk assessment in AS patients, accessible in the rheumatology clinics. The measurements consisted of assessment of basal flow and dynamic PORH tests, which reflect ability of the vasculature to respond to external factors. Dynamic variables such as peak hyperemia and time to peak are more sensitive in establishing endothelial dysfunction. Indeed, the observed relationships were visible particularly in PORH. Basal flow was increased only in reference to TNFi treatment which may be attributed to a greater potency of this factor than disease activity itself. Reversibility of these microvascular changes with clinically effective therapies show that such improvement is aligned with the fact that low disease activity or TNFi therapy reduce inherent CVD risk^34^ and atherosclerosis progression in AS^35,36^. Although LDF and PORH are considered valuable tools to study microcirculation function^16,33^, they have some disadvantages such as indirect blood flow measurement and lack of standardization. Skin vascular bed is also very sensitive to environmental factors which may affect the results and decrease reproducibility. Most important are skin and room temperature and patient’s stress^16^. To overcome this, we controlled environmental temperature before measurements and measured skin temperature before examination which showed no differences between groups (see Supplementary Table S2). Additionally, Bland-Altman plots showed good reproducibility of the performed measurements (see Supplementary Fig. S2). An unexplained issue remains the mechanism of endothelial function improvement after TNFi treatment. LDF assessments were performed at baseline and after 3 months of treatment. Such design made it impossible for us to distinguish whether these effects were chronic or acute. However, the improvement in endothelial function after TNFi treatment is more pronounced and affects post-occlusive as well as basal flow indices, compared to active/inactive disease analysis. Based on that we can speculate that the effect of TNFi can be attributed not only to stabilization of the disease activity and inflammation but also to some other mechanisms – probably neutralization of the direct effects of circulating TNFα.

Nailfold capillaroscopy is also widely used in rheumatology, mainly in diagnosing and monitoring Systemic Sclerosis (SSc). It describes the morphological state of microvasculature, and may be affected by ongoing inflammation. SSc capillary pattern may be sometimes observed in other connective tissue disorders such as polymyositis/dermatomyositis, SLE, undifferentiated connective tissue disease^37^ and Sjögren’s syndrome^38^. We characterized substantial changes in nailfold capillaroscopy between AS patients and healthy controls. Increased occurrence of capillary ramifications and distinct heterogenous shape are hallmarks of ongoing neoangiogenesis. The presence of pericapillary edema may indicate local plasma extravasation or synthesis of extracellular matrix. Microvascular abnormalities in AS are not only limited to functional changes but also affect the morphology, similar to other connective tissue diseases. Neoangiogenesis was not accompanied by other alterations such as capillary loss as in late SSc pattern. Thus, structural changes seem to be secondary to functional impairment which lead to abnormal blood flow, hypoxia and angiogenesis. Interestingly, such morphological abnormalities were already observed in subjects with low disease activity. 3 months of TNFi treatment, in the active disease group, led to a decrease in pericapillary edema, while other parameters were unchanged indicating that while functional changes and edema are reversible, morphological remodeling and number of capillaries is not. While there is no direct evidence of clinical prognostic significance of capillaroscopic measurements in the long term follow up studies, pericapillary edema, that is clearly modified by anti-TNFα treatment, likely reflects systemic inflammation, which may provide a particularly valuable clinical clue.

The limitation of the study are relatively small study groups. However, these were sufficient for achieving sufficient statistical power and were comparable to numerous previous publications^17,36,39^. As treatment was performed according to clinical indication rather than in randomized double blinded fashion, the results should be verified using RCT. Vascular study investigators, were however blinded to the study groups in the initial part of the study, were not part of rheumatology treatment team and did not have access to data regarding treatment of the patients.

In conclusion, this work demonstrates that Ankylosing Spondylitis is associated with changes in microvascular function and morphology, which may explain the increased cardiovascular risk seen in this chronic condition. Microvascular changes were more pronounced in patients with high disease activity and treatment with TNF-α inhibitors significantly improved blood flow in microcirculation and reduced pericapillary edema. This suggests that TNF-α inhibitors may be effective not only in improving the signs and symptoms of AS but also in preventing cardiovascular complications, warranting future randomized outcome trials.

## Patients and Methods

### Subject characteristics and study design

54 AS patients, fulfilling modified New York diagnostic criteria^40^, were recruited consecutively. 28 healthy controls were matched for age, sex, and BMI with no chronic or acute conditions, nor regular medication use (including oral contraceptives). Patients and controls were tested in the same settings in terms of days booked. Matching the population control showed a similar distribution of traditional CV risk factors (Table 1). Power calculation indicated at least 23 patients per group should be studied to observe 30% difference in time to peak hyperemia with 90% power, p < 0.05 (two-sided). Using Bath Ankylosing Spondylitis Disease Activity Index (BASDAI), AS patients were assigned to active AS group (n = 37; BASDAI ≥ 4) or inactive AS (n = 17; BASDAI < 4). Both AS groups were treated with non-steroidal anti-inflammatory drugs (NSAIDs) and had no history of corticosteroid or biological anti-inflammatory therapy.

22 patients with high disease activity who had no observed BASDAI improvement over 3 months, despite use of 2 consecutive NSAIDs, were included in a sub-study, and were treated with TNF-α inhibitor compounds (TNFi) for 3 months. 13 (59%) received adalimumab (40 mg/2 weeks s.c.); 6 (27%) etanercept (50 mg/week s.c.) and 3 (14%) infliximab (5 mg/kg i.v. 0, 2, 6, then 8 weekly). Exclusion criteria were in accordance with the contraindications advised by Assessment of SpondyloArthritis international Society (ASAS)^41^. There were no modifications in concomitant medications throughout the 3 months of TNFi therapy. The study protocol was approved by the Local Ethics Committee of Medical Board of Krakow, Poland (Decision No. 10/KBL/OIL/2013), with all study subjects providing written informed consent. The research was carried out in compliance with the Helsinki Declaration.

### Capillaroscopy

Capillaroscopy evaluated microvessells of the nailfold using a stereo-microscope (Stemi 2000 C, Carl Zeiss, Germany). Capillary visualization was performed using a cold source of light (KL 1500 electronic, Carl Zeiss, Germany), with the use of immersive oil and 100× magnification. The procedure and patient preparation was performed according to previous recommendations^42^. Each test was carried out in a temperature controlled standardized and maintained at 20–22 °C. Study participants followed the specific diet and lifestyle used for our previous flow mediated dilatation studies^43^. Testing was preceded by a 15-minute rest in standardized conditions. Each capillaroscopy assessment was undertaken by two expert investigators who were blinded to the participants’ study group and who were not members of therapeutic team. After the examination final result was obtained based on a consensus meeting. The nailfolds of four fingers on both hands were analyzed (without a thumb). The changes must have been present in at least one finger to be relevant but in the majority of cases were observed in two or more fingers. Morphological parameters were analyzed according to previous observations^44^ with some modifications and defined as follows. Capillary disorganization - irregular capillary distribution and orientation with shape heterogeneity of the loops. Loss of capillaries – less than 5 capillaries in a field of view. Loop enlargements in the efferent, apical, and afferent and efferent part (sometimes called Raynaud loops)- an increase in capillary diameter (homogeneous or irregular) >20 µm. Megacapillaries - enlarged loops with a diameter >50 µm. Capillary ramifications: branching, bushy or coiled capillaries - heterogenous in shape capillaries with visible branches. Capillaries with distinct heterogeneous shape are one of the main morphological features of angiogenesis. Pericapillary edema – foggy or light aspect around capillaries.

### Laser Doppler Flowmetry (LDF)

The microcirculation function was assessed using Laser Doppler Flowmetry (LDF) using the Periflux4001 Master apparatus (Perimed AB, Jarfalla, Sweden). The assessment was based on a study of basal flow and Post-Occlusive Reactive Hyperemia (PORH) test using laser (helium-neon; 780 nm) measuring depth of 0.5–1.0 mm with a probe of standard fiber separation (0.25 mm). Flow parameters obtained allow for sensitive quantification of perfusion and spectrum analysis of blood flow in real time^16^. The probe was stabilized on the dorsal side of distal phalanx of the middle finger. Adhesion of the probe to the skin was controlled by the flowmeter sensor. Each test was carried out in a temperature controlled standardized. Study participants followed the specific diet and lifestyle used for our previous flow mediated dilatation studies^43^. Testing was preceded by a 15-minute rest in standardized conditions. Finger skin temperature was measured using ELLAB CTD-85 system (Copenhagen, Denmark) using the ELLA PRC A probe. The probe was positioned on lateral aspect of the distal phalanx and was stabilized using the pressure cuff of the occlusion test (see Supplementary Fig. S3). Skin temperature measurements are essential to ensure standardization of the assay, and, as expected no differences in skin temperature were observed between studied groups (see Supplementary Table S2).

Following 5 minute basal flow registration, PORH test was performed on the second, third and fourth finger of the hand and average was reported as previously described^45^. Briefly, arterial occlusion was achieved for 5 minutes by inflation of a blood pressure cuff over the proximal phalanx of the finger to supra-systolic pressure values to measure the biological zero. Thereafter, the cuff was deflated and the PORH maximal blood inflow and the time to peak hyperemia were measured. LDF measurements are expressed in arbitrary perfusion units (PU). Microvascular assessment was undertaken by the same expert in microcirculation evaluation who was blinded to the participants study group and who was not a member of therapeutic team. The intra-observer variability was systematically evaluated by serial repeated measurements within a range of possible outputs in a stable disease state. No measurements were rejected for technical reasons. Intra-class correlation coefficients for LDF measurements were: 0.96 (0.90–0.98 CI) for basal flow, 0.93 (0.83–0.97 CI) for peak hyperemia and 0.97 (0.91–0.98 CI) for time to peak. There was no systematic difference between the first and second measurement. Bland-Altman graph for repeatability is shown on Supplementary Fig. S2. The interobserver reliability was calculated based on preliminary experiments using the reliability statistics of the interclass correlation (ICC 1, 2). The ICC coefficients were: 0.946 (0.913–0.987CI) for basal flow, 0.979 (0.948–0.993 CI) for peak hyperemia and 0.975 (0.951–0.991CI) for time to peak.

### Statistical analysis

The statistical analyses were conducted with STATISTICA v12 (StatSoft Inc., Tulsa, OK, USA) for Microsoft Windows v10. Values of measurable parameters were presented as medians with quantiles (Q1–Q3). Box-and-Whisker plots were generated for microcirculation parameters. Statistical comparisons for categorical data were performed using the chi-square or Fisher exact tests and McNemar test on paired nominal data. To compare the statistical significance for continuous data of differences between measurable parameters Mann-Whitney U test and the Wilcoxon rank-sign test were used. The significance level of p < 0.05 was used to test the null hypothesis.

The datasets generated in this study are available from the corresponding author on reasonable request.

## Electronic supplementary material

Supplemental Material
