# Supplementary material for: Microvascular dysfunction in ankylosing spondylitis is associated with disease activity and is improved by anti-TNF treatment
Source: Sci Rep. 2018 Sep 4;8:13205. doi: 10.1038/s41598-018-31550-y (PMC6123474; doi:10.1038/s41598-018-31550-y)
Supplement: Supplementary file 1 — Supplemental Material [file 41598_2018_31550_MOESM1_ESM.pdf]

SUPPLEMENTAL MATERIAL

Microvascular dysfunction in ankylosing spondylitis is associated with disease activity and is improved by anti-TNF treatment

Bogdan Batko, Pawel Maga, Karol Urbanski, Natalia Ryszawa-Mrozek, Agata Schramm-Luc, Mateusz Koziej, Tomasz Mikolajczyk, Eidith McGinnigle, Marta Czesnikiewicz-Guzik, Piotr Ceranowicz, and Tomasz J. Guzik

Table S1. Capillaroscopic parameters in active and inactive Ankylosing Spondylitis group. The number and morphology were evaluated in Ankylosing Spondylitis patients with active (BASDAI ≥4) and inactive (BASDAI <4) disease. The proportions of participants in which the mentioned structures were present. Chi-square test was used.

	Inactive (n=17)	Active (n=37)	P value
Capillary disorganization	17.7%	29.7%	0.54
Loss of capillaries	23.5%	21.6%	0.84
Loop enlargements	23.5%	18.9%	0.73
Megacapillaries	0%	0%	-
Bushy and coiled capillaries	70.6%	78.4%	0.78
Branched capillaries	35.3%	37.8%	0.86
Pericapillary edema	35.3%	51.4%	0.28

Table S2. Mean skin finger temperature after a 15-minute resting period in a temperature controlled room obtained before Laser Doppler Flowmetry assessment. Data are expressed as mean (SD). Student t-test was used. TNFi – TNF-α inhibitor.

	Skin temperature	P value
Active disease	26.52°C (2.50)	0.4
Inactive disease	27.15°C (2.30)	
Healthy controls	27.35°C (2.33)	0.2
Ankylosing Spondylitis	26.64°C (2.20)	
Before TNFi treatment	26.88°C (2.20)	0.7
After TNFi treatment	27.11°C (2.40)	

Figure S1

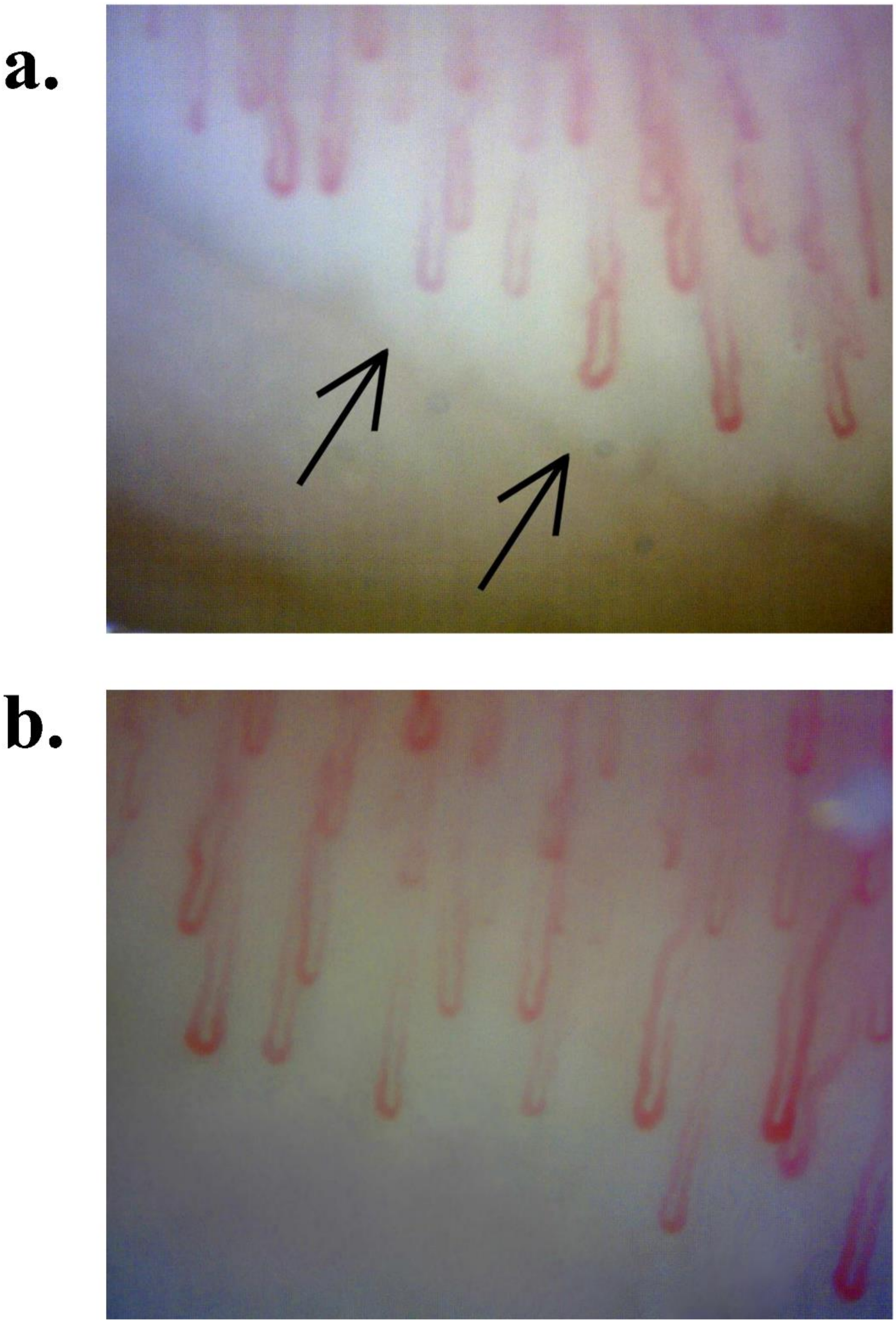

Figure S1. Nailfold capillaroscopy: (**a**) before biologic treatment – visible pericapillary edema; (**b**) after biologic treatment - reduced pericapillary edema. Magnification 100x.

Figure S2

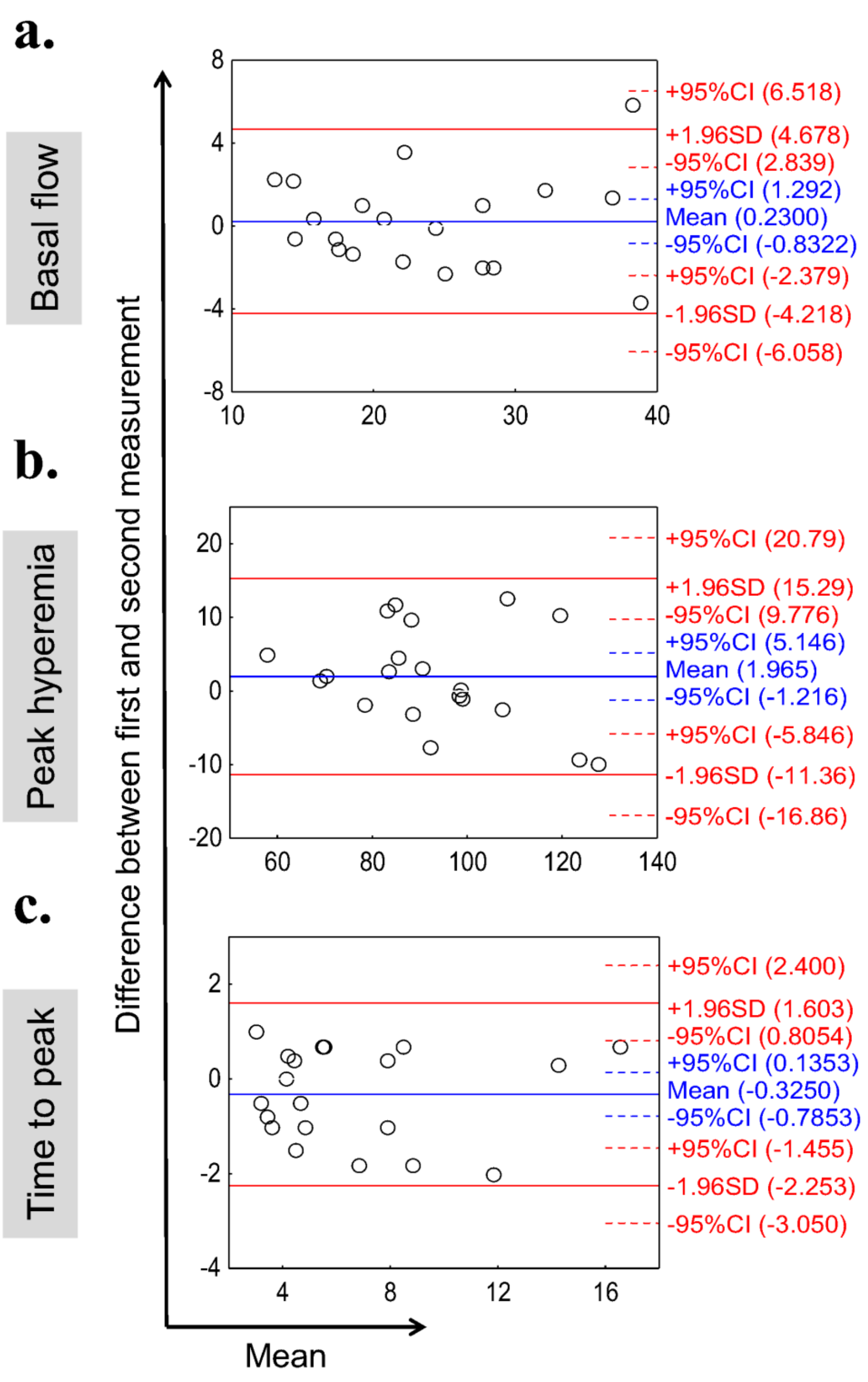

Figure S2. Bland-Altman plots of intraobserver Laser Doppler measurements for: basal flow (**a**), peak hyperemia (**b**) and time to peak (**c**).

Figure S3

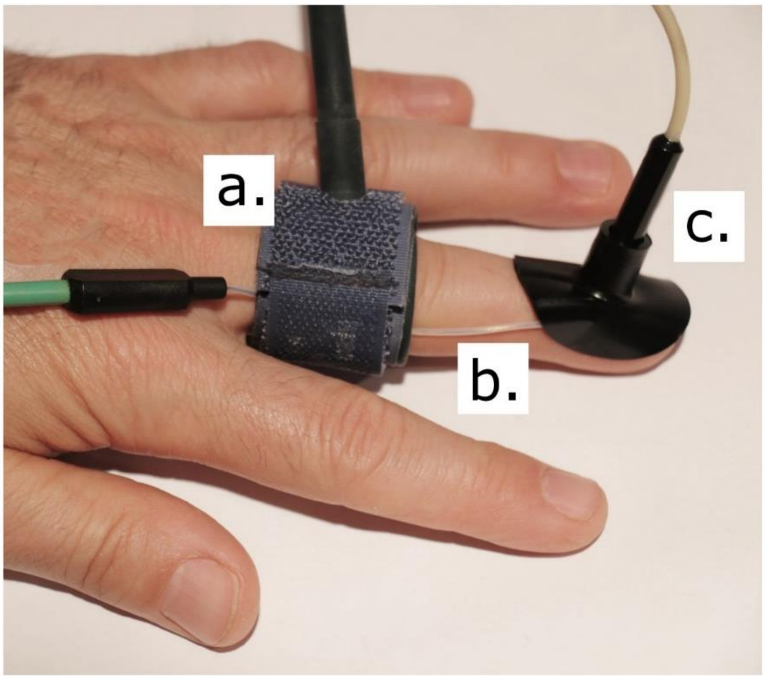

Figure S3. Laser Doppler flowmetry and Post-occlusive Reactive Hyperemia test: (**a**)-cuff pressure; (**b**)-thermometer; (**c**) laser probe and holder.
